# Amyloid Beta Dynamics in Biological Fluids—Therapeutic Impact

**DOI:** 10.3390/jcm10245986

**Published:** 2021-12-20

**Authors:** Thomas Gabriel Schreiner, Bogdan Ovidiu Popescu

**Affiliations:** 1 Faculty of Medicine, University of Medicine and Pharmacy “Carol Davila”, 050474 Bucharest, Romania; bogdan.popescu@umfcd.ro; 2 Neurology Department, University of Medicine and Pharmacy “Grigore T. Popa”, 700115 Iași, Romania; 3 Department of Electrical Measurements and Materials, Faculty of Electrical Engineering and Information Technology, Gheorghe Asachi Technical University of Iasi, 700050 Iasi, Romania; 4 Neurology Department, Colentina Clinical Hospital, 020125 Bucharest, Romania; 5 Laboratory of Cell Biology, Neurosciences and Experimental Myology, ‘Victor Babes’ National Institute of Pathology, 050096 Bucharest, Romania

**Keywords:** Alzheimer’s disease, amyloid beta, amyloidogenic pathway, peripheral sink therapeutic strategy, cerebrospinal fluid sink therapeutic strategy

## Abstract

Despite the significant impact of Alzheimer’s disease (AD) at individual and socioeconomic levels and the numerous research studies carried out on this topic over the last decades, the treatments available in daily clinical practice remain less than satisfactory. Among the accepted etiopathogenic hypotheses, the amyloidogenic pathway theory, although intensively studied and even sometimes controversial, is still providing relevant theoretical elements for understanding the etiology of AD and for the further development of possible therapeutic tools. In this sense, this review aims to offer new insights related to beta amyloid (Aβ), an essential biomarker in AD. First the structure and function of Aβ in normal and pathological conditions are presented in detail, followed by a discussion on the dynamics of Aβ at the level of different biological compartments. There is focus on Aβ elimination modalities at central nervous system (CNS) level, and clearance via the blood–brain barrier seems to play a crucial/dominant role. Finally, different theoretical and already-applied therapeutic approaches for CNS Aβ elimination are presented, including the recent “peripheral sink therapeutic strategy” and “cerebrospinal fluid sinks therapeutic strategy”. These data outline the need for a multidisciplinary approach designed to deliver a solution to stimulate Aβ clearance in more direct ways, including from the cerebrospinal fluid level.

## 1. Introduction

Alzheimer’s disease (AD), a neurodegenerative disease with a huge impact at the public health level, remains a challenge for neurologists in terms of finding an effective therapy [1]. Epidemiologically, it is estimated that over 5 million Americans currently suffer from AD, and predictions for the next few decades show an increase in the prevalence of the disease [2]. In addition, AD is increasingly correlated not only with mortality [3], but also with the financial burden incurred both at the individual level and at the global socioeconomic level [4].

Despite certain already demonstrated risk factors such as age, head injuries, coexisting vascular diseases, infections, and genetic predisposition [5], the cause remains unknown, with no curative treatment available. Over the past 30 years, various studies have tried to explain as completely as possible AD’s etiology and evolution and to use this knowledge for the purpose of developing an effective treatment, leading to several theories [6]. There are two main hypotheses: the cholinergic hypothesis [7] and the amyloidogenic theory [8], although other theories related to neuroinflammation [9], abnormal Tau protein metabolism [10], or free radical damage [11] must also be considered.

Regarding the cholinergic hypothesis, it is based on research initiated in the 1970s related to acetylcholine (ACh), its synthesis and transport at synapse level, and its role in the cognitive process [12]. The synthesis of ACh takes place in cholinergic neurons, starting from choline and acetyl-coenzyme A, the process being performed under the action of the choline acetyltransferase (ChAT) enzyme [13]. Subsequently, ACh is transported by the vesicular acetylcholine transporter to the postsynaptic neuron [14]. At the CNS level, ACh is involved in many higher cognitive processes, such as memory, attention, and learning [15]. Studies on AD have also shown a degeneration of cholinergic neurons [16], explaining the symptoms of the disease, which mainly involve memory loss and impaired cognitive function [17]. Based on these considerations, currently used drug therapies seek to compensate for reduced ACh biosynthesis by inhibiting the ACh degradation enzyme, acetylcholinesterase [18]. Drugs such as donepezil, rivastigmine, or galantamine are currently in use for the symptomatic treatment of AD [19]. In addition, related to ACh, we mention a new class of drugs whose role is to target choline transporter (CHT1) by competitive inhibition, the studies being still in their infancy [20]. CHT1 is a high-affinity choline transporter present on the presynaptic terminal of cholinergic neurons which takes up choline, one of the precursors of acetylcholine [21]. The cholinergic hypothesis is closely related to the amyloid hypothesis (detailed below), as amyloid beta (Aβ) is thought to modulate cholinergic neurotransmission by reducing the choline uptake and subsequently the release of ACh [22]. Studies have demonstrated that as the cholinergic synaptic loss and the amyloid fibril formation are related to Aβ and interaction with ACh enzymes, more attention should be directed toward amyloid precursor protein (APP) metabolism or Wnt signaling [23].

The amyloid hypothesis, one of the oldest and most studied of the AD pathophysiological hypotheses, is currently undergoing a renaissance, with still many clues to offer which might be helpful for the development of new therapies. The link between Aβ accumulation in the brain and the onset of dementia has been observed for decades [24]. However, the fact that abnormal deposition of amyloid beta-sheets was also found in the brains of healthy elderly people poses a question about the causal relationship between Aβ deposits and AD onset [25]. Moreover, the AD classification into two subtypes, depending on the time of onset, although first described in 1997 [26], was supported by subsequent research and is still relevant, representing a starting point for the development of effective therapies. On the one hand, we are talking about early-onset AD (EOAD) which represents only a small percentage [27], while on the other hand the late-onset AD (LOAD) is the sporadic form, and the most common one (95% of cases) [28]. Even though both forms are associated with an imbalance between Aβ production and clearance, resulting in the excessive accumulation of toxic forms of Aβ, the hypotheses claim that the clinical and pathophysiological aspects are different for each form [29].

While the main cause for EOAD dementia would be the increased Aβ production, LOAD is the result of a defective clearance of Aβ [30]. In this context, researchers’ attention has focused in recent years on the dynamics of Aβ between the brain compartment and other biological compartments such as cerebrospinal fluid (CSF) and blood, with respect to the Aβ clearance pathways from the brain. This review aims to present an overview of this very idea, according to which improvement of the deficient Aβ clearance in AD could represent a viable method of treatment. Accordingly, after presenting in detail the structure and function of Aβ and its clinical correlations, the authors provide an overview of the Aβ dynamics between different biological compartments, focusing on the elimination via blood–brain barrier (BBB), which is one of the most important ways of Aβ elimination at the brain level. In the last part, the therapeutic possibilities and the models/hypotheses that can lead to new therapies to increase the Aβ clearance at CNS level are highlighted. The originality of this study, comprising many intensely debated elements about the pathophysiology and therapeutic options of AD, lies in fresh approach to a topic that, despite numerous available studies, still presents unknown facts to researchers.

## 2. Aβ—Production, Structure, Functions, and Therapeutic Correlations

Aβ protein is the result of non-physiological proteolysis of the amyloid precursor protein (APP) [31]. APP, encoded by APP genes located on Chromosome 21, is a Type I transmembrane protein, having a long N-terminal domain and a short cytoplasmic tail [32]. The APP protein family also contains APP Type 1 and 2 proteins (APLP1 and APLP2), which, although very similar in sequence to APP, lack the Aβ domain. In addition, both APLP1 and APLP2 are processed similarly, undergoing ectodomain shedding [33]. In terms of expression and localization, APP and APLP2 are expressed ubiquitously, with particularly high expression in neurons, while APLP1 is found primarily in the nervous system [34].

APP can follow two degradation pathways, one non-amyloidogenic and one amyloidogenic. Under normal conditions, APP is processed via the non-amyloidogenic pathway, first under the action of alpha-secretase (a zinc metalloproteinase), resulting in the sAPPα fragment, a broadly soluble ectodomain of APP [35]. Currently, three members of the ADAM family (a disintegrin and metalloproteinase) are suspected to play a role in this degradation pathway, having α-secretase-like activity: ADAM9, ADAM10, and ADAM17 [36]. Alteration of ADAM17 can alter α cleavage of APP and Aβ generation, α cleavage being abolished in ADAM17-deficient cells [37]. In humans, inhibiting ADAM17 prevented regulated α-secretase activity in human neurons [38]. Regarding ADAM10, its overexpression increases α-cleavage [39], while sAPPα generation was nearly abolished in the neurons of mice with neural ADAM10 conditionally knocked out [40]. sAPPα has multiple roles, demonstrating its action in neuronal plasticity/survival and protection against excitotoxicity in the mature brain [41]. In the early stages of development, sAPPα regulates neural stem cell proliferation [42].

Regarding the amyloidogenic pathway that will end with the production of Aβ, cleavage of APP will be performed under the action of β-secretase. The main β-secretase is BACE1, a membrane-bound aspartyl protease, and considered the rate-limiting factor in Aβ generation from APP [43]. BACE1 was also studied as a potential therapeutic target, with the studies on AD mouse models being worth mentioning, where the deficiency of BACE1 was correlated with an important reduction in Aβ40/42 levels, reduced neuronal loss, and memory deficits [44]. BACE2, a homologue of BACE1, also processes APP at the β site, contributing to AD pathogenesis [45]. Although expressed in neurons substantially lower than BACE1, BACE2 could become a therapeutic target for AD, without the side effects of BACE1 inhibition [46]. Besides BACE1 and BACE2, a lysosomal cysteine protease, cathepsin B, was also proposed as an additional β-secretase, as cathepsin B inhibitors demonstrated mitigation of memory deficit and reduction in beta-amyloid concentration related to AD [47].

**Figure 1:**
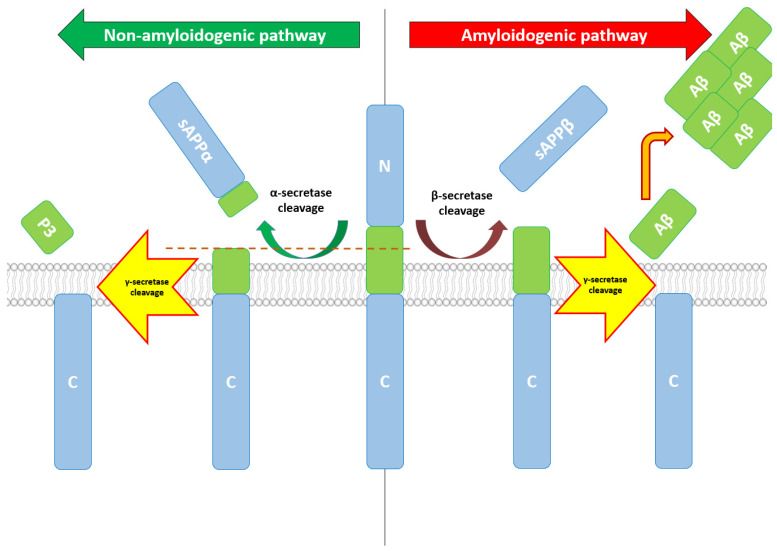
Non-amyloidogenic vs. amyloidogenic pathway.

After the cleavage by either alpha-secretase or beta-secretase, the carboxyl terminal fragments (CTFs) of APP are further altered by gamma-secretase, generating p83 and Aβ, respectively. The gamma-secretase complex comprises at least four components: presenilin (PS, PS1 or PS2), Nicastrin, anterior pharynx-defective-1 (APH-1), and presenilin enhancer-2 (PEN-2) [48]. Several other factors such as CD147 or TMP21/p23 have been proposed as gamma-secretase components, although playing only modulatory roles [49]. Moreover, other enzymes such as caspases (especially caspase-3) can cleave APP in specific positions (position Asp664), releasing a 31 amino acid fragment of APP called C31, with cytotoxic characteristics [50]. For a detailed overview, see Figure 1.

Knowing in detail the amyloidogenic metabolic pathway of Aβ provides essential knowledge for developing new therapies. Β-secretase cleavage, a fundamental step in Aβ production, was already the subject of research when referring to BACE inhibitors (see below), and although clinical trials conducted so far were discontinued for safety reasons, BACE1 remains a well-validated therapeutic target for AD [51].

When referring to the structure of Aβ and its influence on Aβ toxicity, quaternary structures play the major role. Moreover, it has been shown that oligomers are the most toxic/pathogenic structures, compared to monomers or even stable senile plaques [52,53,54]. The formation of oligomers occurs through the nucleation process, which can be a primary process—when two or more monomers are assembled—or a secondary one, when there are already oligomers that facilitate additional nucleation [55]. These two nucleation processes are of therapeutic importance, with conformation-specific antibodies being used experimentally to prevent the formation of toxic oligomers and being a possible treatment modality [56]. However, an important element when talking about the study of oligomers is the differentiation of their influence in vivo compared to in vitro studies. Only Type 2 oligomers (formed by secondary nucleation) share the basic structural features of amyloid fibrils, being found in the vicinity of amyloid plaques. However, regarding the toxic effects at the CNS level, both types of oligomers alter neuronal signaling pathways, leading from varying degrees of synaptic and axonal transport dysfunction to synaptic loss, and neuron death [57]. In order to develop efficient therapies, more research on Aβ oligomers must be conducted in humans, as there are still questions regarding the correlation between temporal and spatial oligomer patterns and neurological dysfunction.

## 3. Aβ Dynamics between ISF, CSF, and Blood

Aβ dynamics, with a focus on the pathways of Aβ elimination from the CNS level, have provoked great interest among researchers in recent years, given that studies increasingly show the link between defective Aβ elimination and LOAD [58]. In addition, if in the past it was thought that most Aβ elimination is performed via BBB, the glymphatic pathway is becoming increasingly important nowadays, with studies showing that astroglial aquaporin-4 (AQP4) channels eliminate significant amounts of extracellular Aβ [59].

Currently, several Aβ purification systems are known at the CNS level (see Table 1), with soluble extracellular Aβ forming the bulk of the total amount of Aβ that has a high mobility between the different biological compartments. The elimination of proteins, and by implication of Aβ, is achieved both at the intracellular level, under the action of proteolytic enzymes, and at the extracellular level, being eliminated in the blood or recirculated in the CSF. The actual percentage contribution of each protein degradation/elimination method remains to be unraveled.

Degradation clearance occurs at the intra- and extracellular level, being supported by different enzyme systems. At the intracellular level, along with protease, Aβ degradation can occur as follows: via ubiquitin–proteasome pathway, via autophagy–lysosome pathway, and via endosome–lysosome pathway [60]. However, these systems are affected in aging or in neurodegenerative diseases, the evidence suggesting that proteasomal activity may be inhibited by Aβ. Dysfunction in the ubiquitin–proteasome pathway appears to promote APP processing, with the formation of increased amounts of Aβ [61].

At the extracellular level, the degradative processes are much simpler, being performed under the action of proteases and phagocytes produced by the astrocyte. However, extracellular proteins can be embedded at the intracellular level, where they will be degraded under the phagocytic action of the microglia or astrocyte [62].

Aβ from the ISF level is cleared mostly via BBB directly into the bloodstream, under the action of specialized transporter systems [63]. It is already known that BBB is one of the three highly selective interfaces between the circulatory system and the CNS, with the role of maintaining cerebral homeostasis, along with the blood–cerebrospinal fluid (CSF) barrier and the arachnoid barrier [64].

Located at the interface between the vascular system and the cerebral substance, more specifically at the level of the endothelium of micro vessels, BBB is not a simple barrier but a complex structure, unique in the human body, its integrity being essential to fulfill its functions correctly [65]. The presence of inflow and outflow transporters is important for Aβ dynamics through BBB. Responsible for the soluble Aβ efflux at the ISF level are LDL receptor (LDLR) family members such as LRP1, and ATP-binding cassette transporters (ABC transporters) [66].

ABCB1 (also known as P-glycoprotein 1 or MDR1) is the main transporter in the ABC transporters family and mediates Aβ efflux directly into the circulation [67]. Studies have further shown a possible association between oxidative stress and downregulation of ABCB1 and other BBB efflux transporters in AD, as AD is considered a chronic inflammatory state with sustained systemic oxidative stress [68].

Another transporter, ABCA1, appears to mediate Aβ efflux in an ApoE-dependent manner. The data are also supported by a meta-analysis [69], which showed that ABCA1 rs2422493 polymorphism was a risk factor for AD, while other mutations may play a role in AD pathogenesis when interacting with ApoE-ε4. Another study showed that ABCA1 controls ApoE lipidation, as ApoE4 promotes ABCA1 trapping in late-endosomes and impairs its recycling to the cell membrane. That is conducive to lower ABCA1-mediated cholesterol efflux activity, a greater percentage of lipid-free ApoE particles, and consequently lower Aβ degradation capacity [70].

LRP1 has been intensively studied in relation to the Aβ accumulation found in AD, its involvement being demonstrated in several stages of the Aβ formation and elimination process. First, there are studies that have shown that LRP1 has the ability to modulate APP processing, influencing Aβ generation. On the one hand, Ref. [71] showed that blocking LRP1 activity will lead to an increased cell surface level of APP and a significant reduction in Aβ synthesis. Other studies demonstrate the opposite, considering that overexpression of the LRP1 C-terminal transmembrane domain can play the role of a competitor for APP when referring to β-secretase and γ-secretase processing, leading to reduced Aβ production [72]. LRP1 is directly involved in the endocytic receptor mediation and trafficking, and in lysosomal degradation of several substrates, with recent works demonstrating receptor-mediated clearance of Aβ through LRP1 in neurons and astrocytes [73]. Besides neural cells, LRP1 acts also at vascular cell level, as recent research confirms LRP1-mediated internalization of Aβ at BBB level in animal models [74]. The same mechanism of active elimination of Aβ from CSF through LRP1 is found at choroid plexus level, forming another important pathway to regulate CSF Aβ concentration [75]. With regard to BBB, LRP1 seems to play an important role in vascular smooth muscle cells and pericytes also. The group of (Kanekiyo et al., 2012) demonstrated the influence of LRP1 suppression in the human brain’s vascular smooth muscle cells, which significantly reduces uptake and degradation of both endogenous and exogenous Aβ [76]. Normal LRP1 activity is also a condition for pericytes’ normal functioning, as these cells regulate BBB integrity and cerebral blood flow by also clearing Aβ through LRP1 mediated pathway [77].

When thinking of Aβ clearance and Aβ equilibrium between different biological fluids or compartments, LRP1 is also important in the peripheral clearance of Aβ. As the peripheral sink therapeutic strategy states, there is a dynamic equilibrium between Aβ pools in the brain and at the peripheries, where Aβ is cleared through peripheral organs such as the liver [78]. However, many studies found no evidence of a therapeutic relevance for AD evolution when Aβ peripheral elimination was increased through different methods, showing that other solutions such as CSF clearance need to be found in order to delay AD evolution [79].

Finally, LRP1 is of therapeutic importance because it can regulate neuronal transmission by direct or indirect interaction with synaptic proteins such as NMDA receptor unit or GluA1 [80]. Studies have shown the LRP1-mediated insulin signaling and glucose uptake regulation in animal models, with reduction in GLUT3 and GLUT4 levels in neurons [81]. In addition, LRP1 regulates the leptin/leptin receptor complex in the hypothalamus, with depletion in neuronal LRP1 being associated with increased food intake, and the subsequent increased risk of obesity and diabetes [82].

Another member of the LDL receptors is LDLR-related protein 2 (LRP2), also known as megalin, which seems to mediate the clearance of Aβ through the BBB when forming a complex with clusterin (also known as ApoJ). Megalin also has a protective function at choroid plexus level, forming a complex with gelsolin, a protein that is produced in the epithelial cells of the choroid plexus [83].

Clearance of Aβ through the BBB is also mediated by α2-macroglobulin (α2M). The interest in the influence of α2M in AD is not new, as earlier reports suggested an association between α2M polymorphisms and an increased risk of neurodegenerative diseases [84,85]. Although newer data does not sustain this correlation [86], elevated levels of α2M were found in the CSF of AD patients (especially males), with clinical significance still to be determined. Besides the interactions with Aβ aggregation and clearance, α2M is involved in other relevant biological processes, such as the interaction with different apoE isoforms [87]. Thus, so far, there is strong evidence suggesting the role of α2M in the inhibition of Aβ peptide aggregation and toxicity, mainly by facilitating its clearance [88]; however, direct individual implication of α2M in the prevention or promotion of neurodegeneration independent of AD remains to be determined.

Insulin-degrading enzyme (IDE) has been proposed to have a role in Aβ clearance through the BBB, which might explain why BBB clearance is sensitive to insulin. IDE along with neprilysin is involved in the intracellular and extracellular degradation of Aβ [89]. Moreover, IDE seems to act specifically on beta-structure forming substrates, its specificity being a potential advantage in therapeutic approaches for AD [90]. Even in a proteolytically inactive form, according to recent research, IDE appears to inhibit Aβ fibrillogenesis through a chaperone-like role [91]. However, the sensitive cerebral Aβ clearance mediated by insulin and IDE cannot be fully explained based only on present known molecules; the involvement of other, still to be detected, transport systems or proteases has also to be taken into consideration.

Aβ transport is bidirectional, including influx into the ISF/CNS, via RAGE (advanced glycosylation end product-specific receptor). RAGE, a 35kD transmembrane receptor of the immunoglobulin super family, was initially identified and characterized for its ability to bind advanced glycosylation end products (AGEs), but also other ligands such as Aβ, with implication in AD pathogenesis [92]. It seems that RAGE is involved through several mechanisms in neurodegeneration. Firstly, Aβ–RAGE interactions are important for the influx of Aβ from blood to the cerebral sector, but also regarding the BBB integrity. RAGE-mediated Aβ cytotoxicity is directed towards the brain’s microvascular endothelial cells, resulting in structural damage of the neurovascular unit [93]. Breakage of the BBB integrity is also induced by Aβ–RAGE interactions that disrupt tight junction proteins via the Ca^2+^-calcineurin (CaN) pathway [94]. Moreover, RAGE is supposed to be also an important contributor to Aβ generation, as increased expression of RAGE in AD enhances the activities of beta- and/or gamma-secretases, stimulating Aβ production [95]. Finally, the role of oxidative stress and sustained chronic neuroinflammation are two other potential mechanisms related to AD pathogenesis. RAGE, acting as a critical player in both initiation and progression of oxidative stress and inflammation, has intricate pathways with Aβ production and clearance at CNS level [96].

Intensive research was conducted during the last decade on alternative ways of ISF clearance to CSF sink. Two main hypotheses have been circulating, namely the perivascular drainage pathway and the glymphatic pathway. As it is still unclear if these two are distinct pathways, or they simply reflect transport along the same pathway captured under differing physiological or experimental conditions, both mechanisms are detailed below. However, recent data are in favor of the non-mutual exclusiveness of the two clearance pathways, as both could be active depending on specific conditions, while anatomical data reveals that they could use different vessel systems [97].

Previous research has demonstrated that perivascular drainage of ISF was directed to the subarachnoid CSF, taking place ubiquitously throughout the brain [98]. Several mechanisms were proposed to explain the directional movement of solutes from ISF to CSF: passive mechanisms such as diffusion, and active mechanisms such as advection and channel- or transporter-mediated facilitative mechanism. More recent studies, where high-performance fluorescence microscopy was used, confirmed that special structures are involved in the bidirectional ISF–CSF fluid exchange. AQP4 channels play a major role, with significantly impaired flow being observed in mice models lacking AQP4; however, other still to be discovered structures also contribute to this pseudo-lymphatic function of this pathway [99]. Several factors influence glymphatic flow, with maneuvers that lead to arterial pulsatility reduction being associated to impaired solute drainage [100]. On the other hand, gravitational factors and circadian rhythm, especially deep stages of sleep, seem to enhance solutes drainage through the glymphatic system [101]. As research is still emerging in this subfield relating to the glymphatic system, its functions and roles in physiological and pathological conditions is not completely known, and we can only assume that the glymphatic system contributes to Aβ clearance from ISF, although its exact share is still to be determined.

From CSF, solutes (including Aβ) enter the final part of the clearance pathway in order to be eliminated in the peripheral circulatory system. Two main modalities were highlighted by physiologic and imagistic studies: clearance through arachnoid villi and blood–CSF barrier (BCSFB) in blood systemic circulation, concomitantly with perivascular and perineural flow into the lymphatic system [102]. Several factors influence CSF absorption by circular and lymphatic peripheral systems, including CSF production, structural integrity of BCSFB and arachnoid villi, and lymphatic flow at perivascular level, with all these factors being affected by pathological conditions. AD alters CSF production in different ways, by impairing the function of the choroid plexus which suffers calcification and fibrosis [64]. The main limitation in the AD context remains the impaired clearance of CSF, based on multiple dysfunctions in CNS solutes elimination mechanisms. Firstly, BCSFB suffers structural changes, with the activity of influx and efflux transporters such as LRP1 and ABCB1 also being significantly altered [103]. Impairment in the circulatory absorption pathway is found also at arachnoid villi level, where outflow resistance is increased in AD patients. Similar to normal pressure hydrocephalus, the proposed mechanism is based on decreased CSF bulk outflow resulting from structural degradation (amyloid deposition and fibrosis) at arachnoid villi level. Regarding lymphatic clearance, it is known that age is a non-modifiable factor that contributes to decrease in absorption activity of the peripheral lymphatic system [104], with the same suppositions being valid also for CSF lymphatic absorption.

Finally, it is observed that during the last decade the paradigm has shifted, as Aβ clearance via BBB pathway has lost some of its importance, with other ways of elimination coming into the spotlight. Moreover, the hype related to the discovery and better understanding of the glymphatic pathway has offered this alternative way of clearance increasing importance in physiological, as also pathological, conditions.

## 4. Current and Future Therapeutic Directions for Aβ Reduction at CNS Level

Starting with the abovementioned protein clearance mechanisms at CNS level, all being to a varying extent affected in AD, several ways to compensate for their impairment have been examined, including increasing Aβ clearance through alternative mechanisms (see Table 2).

A first way to compensate for the decreased Aβ clearance at CNS level is to inhibit its production. Based on theoretical knowledge about the amyloid cascade, one of the first suggested treatments, no longer valid today, consisted in inhibiting BACE. Studies on murine models were first performed, with several working groups succeeding in synthesizing molecules that had the ability to penetrate the BBB and selectively inhibit BACE1 and BACE2. The results showed a decrease in Aβ brain accumulation [105], but without a real improvement in memory and behavioral deficits [106]. Despite extensive research performed in several mouse models such as double transgenic mice (APP23 × PS45)—[107] or Tg2576 transgenic mice—[108], none of the major BACE1 inhibitors tested were documented to show behavioral effects in AD animal models. Clinical trials in humans followed, involving the administration of different compounds (Verubecestat, Atabecestat, Umibecestat) among various groups of patients in different stages of evolution (preclinical/early AD—[109]; mild AD and mild-to-moderate AD—[110]), with most of them being stopped in the early stages considering the lack of clinical benefit, compounded also, however, by significant side effects such as drug worsened cognition, brain atrophy, and weight loss [111]. Given the results, expert opinion considers the failure of this therapy a sufficient reason to forget this approach and focus on other directions/points of interest on the amyloidogenic pathway.

A completely opposite approach to the Aβ production inhibition is to stimulate the elimination of Aβ from the CNS and/or Aβ enzymatic degradation. Therapeutic clearance of Aβ involves several strategies, but the most researched remains immunotherapy. The principle of immunotherapy consists in activating the immune system, the method’s final role being the degradation of Aβ. There are two ways to achieve this goal: active immunotherapy, in which the production of anti-Aβ antibodies is stimulated, and passive immunotherapy, in which antibodies are administered directly [112]. There are currently several antibodies available, including for clinical use, that have been shown to reduce cerebral Aβ load in both basic research and human clinical trials. However, there is a lack of correlation between the neuropathological benefits and the expected clinical improvement, due to several suspected reasons: the efficiency of antibodies is maximum on Aβ oligomers that have not yet aggregated in the form of senile plaques, thus requiring administration in the prodromal/early stages; there are also other factors such as age or ApoE which influence the behavior of Aβ oligomers and the interaction with possible antibodies [113].

Even if the research carried out so far has not brought the desired results, the mechanisms underlying the process by which anti-Aβ antibodies remove Aβ from the brain are key in the design of new therapeutic approaches. Two separate mechanisms have been proposed, which are not mutually exclusive. On the one hand, microglia with their phagocytic capabilities would capture the antigen-antibody complex, once it is formed at the ISF level. On the other hand, clearance also occurs in the periphery. There are data which support “the peripheral sink therapeutic strategy” and show that the elimination/decrease in the peripheral level of Aβ leads to a reduction in the cerebral Aβ level [114]. The validity of this method has been questioned by subsequent studies [115]; however, many variables remain under discussion, given that results on different mouse models have delivered different, sometimes opposite, conclusions. For example, mice with the Dutch and Iowa mutations tend to accumulate more Aβ in the brain, as Aβ is in this case a poor substrate for LRP transporter. Consequently, peripheral sink therapeutic strategy remains inefficient, with low blood Aβ levels being correlated to high, unmodifiable brain/ISF Aβ concentration [116]. Even in human studies, the results were not as desired, a recent meta-analysis showing that, despite being well tolerated, peripherally administered anti-Aβ immunotherapy does not significantly improve the primary outcome measures. In addition, it must be considered that the peripheral Aβ pool is not only the result of the passage of Aβ from the brain to the peripheral circulation, but also of Aβ being produced in the periphery from cells other than neurons (hepatocytes, platelets) [117].

Based on these assumptions, alternative strategies have been tried, which consist in reducing the Aβ blood level. Among them, we mention plasmapheresis, with positive preliminary results in a phase 2b/3 trial in a group of mild-to-moderate AD patients, where a decrease in cognitive and functional decline was observed [118]. In addition, based on the “peripheral sink therapeutic strategy”, Aβ peripheral clearance was tried by peritoneal dialysis [119] and hemodialysis [120], with satisfactory preliminary results.

However, a much more direct way to eliminate Aβ remains to be explored, more precisely at the CSF level. Knowing the equilibrium between ISF and CSF, as well as the shortcomings of peripheral Aβ clearance methods, the approach based on the “CSF sink therapeutic strategy” seems much more natural and having better therapeutic potential. The decrease in Aβ concentration at the CSF level will determine the balancing of the Aβ concentrations between the communicating fluidic compartments CSF–ISF, as also the decrease of the soluble Aβ concentration at the ISF level. Hypothetically, the elimination of soluble Aβ will also mobilize the insoluble, initially difficult to mobilize, Aβ from the stocks, possibly even influencing the degradation of senile plaques. However, this hypothesis must be validated by studies on human and murine models in the near future. There is nowadays the possibility of accessing the CSF compartment by using implantable biocompatible devices that are based on nanotechnologies. Several technologies such as unidirectional and bidirectional ventriculoperitoneal derivation and lumboperitoneal shunting are already being employed in other pathological conditions, e.g., hydrocephalus [121]. Implantable intrathecal pumps are more advanced biomedical devices that have seen an increase in interest during the last year, as they help in delivering medications directly into the lumbar subarachnoid space [122]. With an inspirational starting point linked to current existing technologies, a very successful future direction could be the development of similar medical devices tailored for Aβ clearance as at least an adjuvant therapeutic approach.

Finally, we present another potentially effective method, although it has not yet been adopted in current clinical practice, whose mechanism of action is based on amyloid cleavage. Among the enzymes capable of degrading physiologically relevant peptides, neprilysin has attracted interest because of its ability to degrade both amyloid beta peptides 1–40 and 1–42 rapidly and efficiently [123]. Neprilysin, also known as neutral endopeptidase (NEP), is an integral Type II membrane-bound zinc-dependent peptidase normally expressed by a variety of tissues [124]. NEP has a quite broad substrate specificity, having however a stronger preference for peptides such as enkephalins, tachykinins, and natriuretic peptides [125]. By cleaving peptides at the N-terminal side of hydrophobic amino acid residues, NEP is responsible for the degradation of a variety of physiological substrates, limiting their activity. As regards AD, NEP is considered a potential therapeutic strategy for the prevention and treatment of the disease, being able to degrade Aβ at brain level. Research in animal models has revealed that manipulation of the levels of brain NEP has a significant effect on Aβ levels. Numerous study groups have shown that NEP gene transfer reduces human amyloid pathology in transgenic mice [126,127,128,129,130]. These promising preliminary results have led to extensive research on this topic, with two directions being relevant here. Firstly, in order to create a potent therapeutic tool for AD, the activity and specificity of NEP towards Aβ need to be better understood and improved. In this regard, the works of [131,132] bring new insights at the molecular level of the Aβ degradation by NEP, paving the way to creating a NEP mutant enzyme with higher efficiency in degrading Aβ in vivo. Secondly, Ref. [133] assessed the therapeutic potential of the intracerebral delivery of neprilysin as a dynamically controllable Aβ(40)-degrading therapeutic strategy for AD. Recent studies [134,135] concentrate on the improvement of NEP delivery across the BBB using novel transport systems, as peripheral administration of NEP does not affect central levels of Aβ. Moreover, immunogenicity may be a side effect in some cases [115].

## 5. Conclusions

Although incomplete and lacking full explanation of the etiopathogenesis of AD, the amyloidogenic hypothesis remains one of the most important theories for understanding the disease and developing potential therapies [136]. In addition, continuous discoveries in recent years regarding Aβ dynamics, such as the growing importance of the role of the glymphatic system in the clearance of molecules involved in AD pathogenesis [137], are challenging the classical paradigms. Moreover, acceptance of the existence of two forms of AD, EOAD and LOAD, with the latter being predominant and based on impaired Aβ clearance, suggests that in order to be successful in the treatment of AD, future approaches must focus on ways that increase Aβ elimination from the CNS; or, as the direct elimination from ISF is currently unfeasible, new theories such as the “peripheral sink therapeutic strategy” or the “CSF sink therapeutic strategy” become the theoretical basis for the development of new technologies that will be able to eliminate Aβ directly from circulation and CSF level, ultimately reducing the cerebral Aβ concentration. This therapeutic path remains open and of great interest, requiring interdisciplinary research on biocompatible nanostructures in order to come up with a feasible solution first on murine models, and later with satisfactory results in daily clinical practice.

## Figures and Tables

**Table 1:** Aβ clearance pathways from CNS.

Clearance Pathway	Direction/Biological Compartment	Key Players	Alterations in Pathological Conditions (AD)
Blood–brain barrier	ISF to peripheral circulation	LRP1, LRP2, ABCB1, ABCA1, α2-macroglobulin, IDE, ApoE, RAGE	Reduced efflux Increased RAGE-influx
Intracellular degradation	Microglia, Astrocyte	ubiquitin–proteasome pathway, autophagy–lysosome pathway, endosome–lysosome pathway	Reduced
Extracellular degradation	ISF	Proteases, phagocytosis (microglia/astrocyte uptake)	Reduced
Perivascular drainage	ISF to CSF	Diffusion Transporter-mediated active mechanism	Reduced diffusion
Glymphatic system	ISF to CSF	Bulk flow AQP4 Sleep	Unknown (probably reduced)
CSF absorption	CSF to peripheral circulation CSF to peripheral lymphatic system	Brain–CSF barrier Arachnoid villi Lymphatic absorption	Reduced flow via brain-CSF barrier and arachnoid villi

AD—Alzheimer’s disease; ISF—interstitial fluid; CSF—cerebrospinal fluid.

**Table 2:** Alzheimer’s disease—therapies with focus on the amyloidogenic pathway.

Type of Treatment	Pathophysiological Mechanism	Use/Efficiency
BACE inhibitors	Inhibit BACE1 and BACE2 in order to minimize Aβ production	Inefficient Several important adverse effects (brain atrophy, weight loss) Currently not recommended
Aβ monoclonal antibodies	Immunotherapy (Antigen-antibody complex)—favors Aβ elimination	Inconsistent results Recently approved Adacunumab for clinical use
Aβ vaccine	DNA vaccination for anti-Aβ immunotherapy	Phase III clinical trials ongoing
RAGE inhibitors	Inhibition of RAGE Inhibition of Aβ influx to CNS Reduction in oxidative stress and neuroinflammation	Azeliragon tested in phase 2/3 trials—missed endpoints Research in progress
Plasmapheresis	Reduction in Aβ peripheral level The “peripheral sink therapeutic strategy”	Positive preliminary results
Peritoneal dialysis	Reduction in Aβ peripheral level The “peripheral sink therapeutic strategy”	Positive preliminary results
Implantable intrathecal pumps	Reduction in Aβ CSF level The “CSF sink therapeutic strategy”	Near future approach
Aβ cleavage	Degradation of Aβ at both CNS and peripheral level	Intracerebral delivery of neprilysin—positive preliminary results Peripheral delivery of neprilysin—no impact on Aβ at brain level
